# Validity and reliability of the Turkish version of the Innsbruck RBD-9 diagnostic inventory (IRBD-9-TR)

**DOI:** 10.1055/s-0044-1800816

**Published:** 2025-01-28

**Authors:** Kezban Aslan-Kara, Ayşın Kısabay Ak, Ayşegül Şeyma Sarıtaş, Hikmet Yılmaz, Kübra Mehel Metin, Burcu Gökçe Çokal, Kadriye Ağan, Murat Aksu, Utku Oğan Akyıldız, Aylin Bican Demir, Betül Çevik, Ahmet Yusuf Ertürk, Derya Karadeniz, İbrahim Öztura, Gülin Sünter, Selma Tekin, İrsel Tezer, Deniz Tuncel Berktaş, Nazlı Totik, Gülçin Benbir Şenel

**Affiliations:** 1 Cukurova University Medical Faculty, Department of Neurology, Division of Clinical Neurophysiology, Adana, Turkey.; 2 Celal Bayar University Medical Faculty, Department of Neurology, Manisa, Turkey.; 3 Ankara Training and Research Hospital, University of Health Sciences, Department of Neurology, Ankara, Turkey.; 4 Marmara University Medical Faculty, Department of Neurology, Istanbul, Turkey.; 5 Acibadem University Atakent Hospital Medical Faculty, Department of Neurology, Istanbul, Turkey.; 6 Adnan Menderes University Medical Faculty, Department of Neurology, Aydın, Turkey.; 7 Bursa Uludag University Medical Faculty, Department of Neurology, Bursa, Turkey.; 8 Tokat Gaziosmanpasa University Medical Faculty, Department of Neurology, Tokat, Turkey.; 9 Dokuz Eylul University Medical Faculty, Department of Neurology, Izmir, Turkey.; 10 Istanbul University-Cerrahpasa Faculty of Medicine, Department of Neurology, Division of Clinical Neurophysiology, Istanbul, Turkey.; 11 Pamukkale University Medical Faculty, Department of Neurology, Denizli, Turkey.; 12 Hacettepe University Medical Faculty, Department of Neurology, Ankara, Turkey.; 13 Kahramanmaras Sutcu Imam University Medical Faculty, Department of Neurology, Kahramanmaras, Turkey.; 14 Cukurova University Medical Faculty, Department of Biostatistics, Adana, Turkey.

**Keywords:** REM Sleep Behavior Disorder, Reproducibility of Results

## Abstract

**Background** Isolated rapid eye movement (REM) sleep behavior disorder (iRBD) is characterized by loss of the normal atonia of REM sleep accompanied by repetitive motor and behavior phenomena of dream content.

**Objective** To evaluate the reliability and validity of the Turkish version of the original form of the Innsbruck Rapid Eye Movement Sleep Behavior Disorder Diagnostic Inventory (IRBD-9) scale (IRBD-9-TR) and ensure that this screening test can be easily used in the Turkish language.

**Methods** The present is a multicenter and prospective study involving 184 patients: 51 with iRBD and 133 healthy controls. The iRBD patients were not diagnosed before submitted to video polysomnography (vPSG) and filling out the IRBD-9-TR.

**Results** The optimal cut-off value for the IRBD-9-TR symptom score was of 0.28, with a sensitivity of 0.941 and a specificity of 0.947, and 94.4% of the patients were correctly diagnosed. The rotated factor loadings for the diagnostic accuracy of each individual question showed that the short version of the IRBD-9-TR (questions 1, 2, 3, 6, and 8) presented higher specificity and excellent discrimination of iRBD patients from healthy controls. The Cronbach's α coefficient for the symptom section of the IRBD-9-TR was of 0.857, and the Kappa coefficient, of 0.885.

**Conclusion** The short version of the IRBD-9-TR presents good validity and reliability to be used as a screening test to assess iRBD patients. It is convenient and potentially useful in both outpatient clinical and epidemiologic research settings.

## INTRODUCTION

Isolated rapid eye movement (REM) sleep behavior disorder (iRBD) is one of the parasomnias characterized by repetitive motor phenomena that overlap with dream content during REM sleep. 1 The physiopathology of iRBD was first described by Jouvet and Delorme, developed bilateral, symmetrical mediodorsal pontine tegmental lesions in cats, and defined “paradoxical sleep without atonia”, in 1965. 2 3 The latest diagnostic criteria were defined by the American Academy of Sleep Medicine (AASM) in the third edition of the International Classification of Sleep Disorders (ICSD-3). 4

Epidemiological studies on iRBD are very limited due to the high cost of video polysomnography (vPSG) and the time-consuming methodology. In prevalence studies, the rate of IRBD was of 5.2% when screening with a validated single-question form, 5 6 while it was found to be as low as 0.38% to 1.12% with the evaluation of the patients through vPSG. 7 8 In prodromal Parkinson's disease (PD) screening studies, different investigators have confirmed the presence of iRBD in 65% to 75% of the patients. 6 9

It is now accepted that iRBD is an α-synucleinopathy, and a prodromal feature of PD, dementia with Lewy bodies or multiple system atrophy. 1 Therefore, the identification of idiopathic RBD or iRBD is of crucial importance to make an appropriate and early diagnosis, to follow-up the patients for the phenoconversion to neurodegenerative diseases, and to provide appropriate support and neuroprotective treatments. 10 11

Questionnaires are very useful and easy to apply to screen high numbers of patients at risk for RBD and to decide which patients should undergo a more detailed investigation, such as through vPSG. There are only limited numbers of RBD-screening questionnaires, and only one questionnaire, the Rapid Eye Movement Sleep Behavior Disorder Screening Questionnaire (RBDSQ), has been validated in the Turkish language. 12 The Innsbruck Rapid Eye Movement Sleep Behavior Disorder Diagnostic Inventory (IRBD-9) 13 was designed as a screening test that can be easily applied in clinical settings, and it has been reported to present high specificity and sensitivity to assess both the presence and frequency of iRBD-specific symptoms. 13

The aim of the present study was to investigate the validity and reliability of the Turkish version of the IRBD-9 scale (IRBD-9-TR) with the goal of enhancing the ease of its use in the routine clinical practice and for population-based epidemiological studies.

## METHODS

The current is a multicenter and prospective study. After approval from the institutional Ethics Committee (23.05.2022/122; decision no: 46), the proposal of the study was sent to all accredited sleep centers, which have at least one sleep expert with a Turkish national certificate. A total of 13 accredited sleep centers throughout Turkey participated and contributed to the study. The RBD patients were recruited from sleep centers between September 2022 and April 2023.

### Procedure and subjects

The initial step in adapting the scale to Turkish was to consult with the scale's developer, Dr. Högl, and the MAPI (Online Support of Clinical Outcome Assessments: https://eprovide.mapi-trust.org/ ) group. The requisite permission to translate the instrument into Turkish was obtained from the Turkish Sleep Study Group (MULA Reference: Turkish Sleep Study Group_TR_431114_MULA_20220608_FE).

The English version of the scale was translated into Turkish language by three blinded neurologists certified in English. The three Turkish versions of the test were translated back into English language, at weekly intervals by another medical doctor with native level of proficiency in English and Turkish languages. The consistency between the Turkish and English versions was analyzed. Then, the Turkish version was examined by a Turkish linguist regarding meaning and grammar, and it was made ready for use in “validity” and “reliability” studies ( Table 1 ). To ensure validity, the Turkish version of the RBDSQ 12 (RBDSQ-T) was used as a reference.

**Table 1:** Items evaluated for the IRBD-9 (Turkish translation from English)

Questions	
**Turkish**	
**1**	Uykunuzda, şiddet veya saldırganlık içeren rüyalar görür müsünüz? (kendinizi korumak zorunda kaldığınız rüyalar ve benzeri)
**2**	Uykunuzda çığlık atıp, hakaret ediyor veya küfür ediyor musunuz? (Not: normal uyku konuşması dâhil değildir)
**3**	Uykunuzda hareket ediyor ve ara sıra “sıçrama-silkelenme” veya daha aşırı hareketler yapıyor musunuz?
**4**	Erişkin olduğunuzdan beri, uykunuzda yatağınızı terk edip odadan çıktığınız oldu mu?
**5**	Uyurken hiç yataktan düştünüz mü?
**6**	Uyurken kendinizi veya eşinizi yaraladınız mı veya neredeyse yaralayacak oldunuz mu? (ör: istem dışı vurdunuz mu?)
**7**	Uykuya dalarken komodinin üzerinde olan eşyaları uyandığınızda yerde bulduğunuz oldu mu? (örneğin çalar saat, cep telefonu vb.)
**8**	Yukarıda tanımlanan uykudaki hareketler, ara sıra veya sürekli olarak gördüğünüz rüyalarınızın içeriği ile aynı mı? (2, 3, 6 maddeler)
**9**	Yüksek sesle ve düzensiz bir şekilde horlar mısınız veya uyurken düzensiz nefes aldığınızı biliyor musunuz?
**English**	
**1**	Do you dream of violent or aggressive situations (such as having to defend yourself)?
**2**	Do you scream, insult, or curse during your sleep? (Note: this does not include normal sleep talking.)
**3**	Do you move out of your sleep and occasionally perform ‘‘flailing’' or more extensive movements?
**4**	Have you left your bed and have you gone out of the room during your sleep since entering your adulthood?
**5**	Have you ever fallen out of bed while you were sleeping?
**6**	Have you ever injured or nearly injured yourself or your bed partner while you were sleeping?
**7**	Have you ever found items that were placed on the bedside table when falling asleep lying on the floor when you awakened (alarm clock, mobile phone, etc.)?
**8**	Are the above-described movements out of your sleep occasionally or always in line with the content of your dreams (items 2, 3, 6)?
**9**	Do you snore loudly and irregularly, or do you know you have an irregular breathing during your sleep?

Abbreviation: IRBD-9, Innsbruck Rapid Eye Movement Sleep Behavior Disorder Diagnostic Inventory.

Patients admitted to the outpatient sleep clinics during the study protocol were prospectively and consecutively evaluated. We included iRBD patients with a mean age of 64.90 ± 7.69 (range: 49–82) who had episodes such as repetitive complex motor movements, shouting, and vocalization during sleep, who had had these episodes during REM sleep documented through vPSG, and who met the diagnostic criteria according of the ICSD-3. 4 Patients with RBD secondary to alcohol, drug, psychiatric illness, narcolepsy, paraneoplastic, PD and other neurodegenerative disorders, as well as other α-synucleinopathies, and patients who could not undergo the study procedures were excluded. Healthy subjects were also prospectively and consecutively selected, and they all underwent vPSG to exclude any other sleep disorders. Moreover, healthy subjects with other associated medical conditions that might interfere with sleep or iRBD, such as epilepsy or the use of serotonin reuptake inhibitors, were excluded.

### Polysomnographic investigations

Full-night vPSG examinations were recorded and analyzed by the sleep experts based on criteria of the AASM Manual for the Scoring of Sleep and Associated Events. 14 In the electromyographic (EMG) recordings, the amplification of the activity of the chin and extremity electrodes (tibialis anterior muscle) was performed according to AASM standard data, with an amplification of 5 μV/mm, low (10 Hz) and high (100 Hz) filter frequencies, and a sampling frequency of 500 Hz.

The vPSG parameters included time in bed (TIB), total sleep time in bed, sleep efficiency (SE), total sleep time (TST), sleep latency (SL), latency of REM sleep, the percentages of wakefulness (W) and other sleep stages (N1, N2, N3, REM sleep), wake time after sleep onset (WASO), the apnea-hypopnea index (AHI), and the Periodic Limb Movement Index (PLMI).

Rapid eye movement sleep without atonia (RSWA) was defined as sustained (tonic) and phasic EMG activity scored from chin and/or from the anterior tibial muscles EMG channels. Tonic EMG activity was defined as an increase in the amplitude of the EMG electrode greater than the minimum amplitude demonstrated in NREM sleep and lasting for at least 50% of the duration of an epoch. Phasic EMG muscle activity was defined as bursts of transient muscle activity in at least 5 out of 10 (50%) 3-second mini-epochs, with each burst lasting for 0.1 to 5.0 seconds and at least 4 times as high in amplitude compared to the background EMG activity. 14

### Innsbruck REM Sleep Behavior Disorder Inventory

The IRBD-9-TR items were scored using the calculation in the study by Frauscher et al. 13 In the first section, the answers to questions are “yes” (meaning the symptom is present), “no” (meaning the symptom is absent), or “don't know.” “Don't know” responses were counted as missing values in the analysis. The RBD symptom score was calculated by dividing the total number of “yes” answers by the total number of questions. As a result, the IRBD symptom scores varied from 0 (the lowest) to 1 (the highest).

In the second section of the inventory, the frequency of occurrence of symptoms in the previous year was assessed on a scale from never to very frequent (more than twice per week), with the options being “never”, “rare” (once to a few times per year), “occasional” (once to a few times per month), and “frequent” (1 to 2 times per week). “Never” was as assigned a score of 0, “rare” score of 1, “occasional” score of 2, “frequently” score of 3, and “very frequent” score of 4. The scores on all items were added and divided by the total number of questions (RBD frequency score). As a result, the RBD frequency scores varied from 0 (the lowest) to 4 (the highest).

### Statistical analysis

Depending on whether or not the statistical assumptions were met, the Student's *t* -test or the Mann-Whitney U test were employed to compare the continuous variables of two groups. Pearson's correlation analysis was used for assess the correlation between two variables. The results were reported as mean ± standard deviation values when appropriate. The reliability of the IRBD-9-TR was evaluated using internal consistency (calculated through the Cronbach's α coefficient). The sensitivity and specificity for various cut-off values were determined and shown using a receiver operating characteristic (ROC) curve. The area under the curve (AUC) was used to determine the diagnostic value of the IRBD-9-TR. 15 The Kappa coefficient was employed to assess the degree of agreement between the RBDSQ-T scale, which has been demonstrated to be valid (and thus considered the gold standard), and the IRBD-9-TR. According to Landis and Koch, 16 Kappa values < 0 indicate “poor” agreement; from 0.00 to 0.20, “slight” agreement; from 0.21 to 0.40, “fair” agreement; from 0.41 to 0.60, “moderate” agreement; from 0.61 to 0.80, “substantial” agreement; and from 0.81 to 0.99, “almost perfect” agreement. A Kappa coefficient of 1 indicates complete agreement.

The Cronbach's α coefficient was determined to assess the questionnaire's reliability. Values of the AUC > 0.70 were considered sufficient, and Cronbach's α > 0.7 was regarded as satisfactory. 17 18 The scale's structural validity was evaluated using exploratory factor analysis.

The Kaiser–Meyer–Olkin (KMO) test is used to determine how well the components explain one another in terms of partial correlations among variables. The KMO values range from 0 to 1; values near 1.0 are considered outstanding, while those < 0.5 are rated unsatisfactory. If the value is < 0.5, the factor analysis results will most likely be ineffective for data analysis. The Bartlett's test of sphericity is used to determine whether the correlation matrix is an identity matrix. If there is an identity correlation matrix, the variables are unrelated, making the factor analysis unsuitable. 19 Values of *p* < 0.05 were considered statistically significant, and the statistical analyses were performed using IBM SPSS Statistics for Windows (IBM Corp., Armonk, NY, United States) software, version 20.0.

## RESULTS

### Sample characteristics and total scores on the IRBD-9-TR

The sample of the present study was composed of 184 patients, 51 with iRBD and 133 healthy controls, who met the inclusion criteria; their mean age was of 49.98 ± 14.24 years and, regarding gender, there were 45% of female and 55% of male subjects. As for the mean symptom score on the IRBD-9-TR, it was of 0.21 ± 0.25 points (ranging between 0 and 1 points) for the whole sample, but it was significantly higher among iRBD patients compared to the healthy subjects ( Table 2 ).

**Table 2:** Descriptive statistics of demographic and clinical data of the study sample

		iRBD ( *n* = 51)	Healthy subjects ( *n* = 133)	*p*
Gender ^**a**^	Male ( *n* = 91)	32 (62.7)	59 (44.4)	0.039
	Female ( *n* = 93)	19 (37.3)	74 (55.6)	
Age (years) ^**b**^		64.90 ± 7.69	43.45 ± 12.68	< 0.001
IRBD-9-TR total symptom score ^**b**^		0.56 ± 0.20	0.07 ± 0.12	< 0.001
RBDSQ-T total score ^**b**^		8.67 ± 1.87	1.41 ± 1.74	< 0.001
RBD duration (months) ^**b**^		50.18 ± 69.90	NA	−
PLMI ^**b**^		15.97 ± 20.36	8.57 ± 12.85	0.374
RSWA ^**a**^	None	0 (0.0)	131 (98.5)	< 0.001
	Chin	0 (0.0)	1 (0.8)	
	Leg	2 (3.9)	1 (0.8)	
	Both of chin and leg	49 (96.1)	0 (0.0)	
Duration of leg RSWA ^**b**^ (seconds)		61.58 ± 220.90	1.41 ± 13.87	< 0.001
Duration of chin RSWA ^**b**^ (seconds)		156.26 ± 482.56	3.01 ± 34.24	< 0.001

Abbreviations: iRBD, isolated rapid eye movement sleep behavior disorder; IRBD-9-TR, Turkish version of the Innsbruck Rapid Eye Movement Sleep Behavior Disorder Diagnostic Inventory; PLMI, Periodic Limb Movement Index; NA, not applicable; RBD, rapid eye movement sleep behavior disorder; RSWA, rapid eye movement sleep without atonia; RBDSQ-T, Turkish version of the Rapid Eye Movement Sleep Behavior Disorder Questionnaire..

Notes: ^**a**^ Data expressed as n (%); ^**b**^ data expressed as mean ± standard deviation values.

### Construct validity

Exploratory factor analysis is applicable for the IRBD-9-TR inventory as the *p* -value for the Bartlett's test of sphericity was < 0.001, and the value of KMO measure of sampling adequacy was of 0.85. Table 3 shows the rotated factor loadings for items with values > 0.5, and it illustrates the varimax-rotated two-factor solution for the IRBD-9-TR. With the item loading ranging from 0.61 to 0.92, the two-factor model explained a significant portion of the scale variation (that is, 60.5%). Five items (questions 1, 2, 3, 6, and 8) were loaded on factor I, and the rest of the items (questions 4, 5, 7, and 9) were loaded on factor II.

**Table 3:** Rotated factor loadings for the symptom section of the IRBD-9-TR

Scale item	Factor I	Factor II
1	0.91	
2	0.92	
3	0.81	
4		0.78
5		0.61
6	0.75	
7		0.61
8	0.88	
9		0.61

Abbreviation: IRBD-9-TR, Turkish version of the Innsbruck Rapid Eye Movement Sleep Behavior Disorder Diagnostic Inventory.

The mean scores for the two factors were further evaluated: in the comparison between the RBD patients and the healthy subjects, the RBD patients scored significantly higher on factors I and II. The mean scores of the groups for both factors are presented in Table 4 .

**Table 4:** Comparison of the mean scores of the two factors from the exploratory factor analysis

Symptom score	RBD ( *n* = 51): mean ± SD	Healthy subjects ( *n* = 133): mean ± SD	*p*
Factor I (questions 1, 2, 3, 6, and 8)	0.78 ± 0.21	0.04 ± 0.14	< 0.001
Factor II (questions 4, 5, 7, and 9)	0.29 ± 0.28	0.11 ± 0.16	< 0.001

Abbreviations: RBD, Rapid Eye Movement Sleep Behavior Disorder; SD, standard deviation.

### Internal consistency

**Figure 1:**
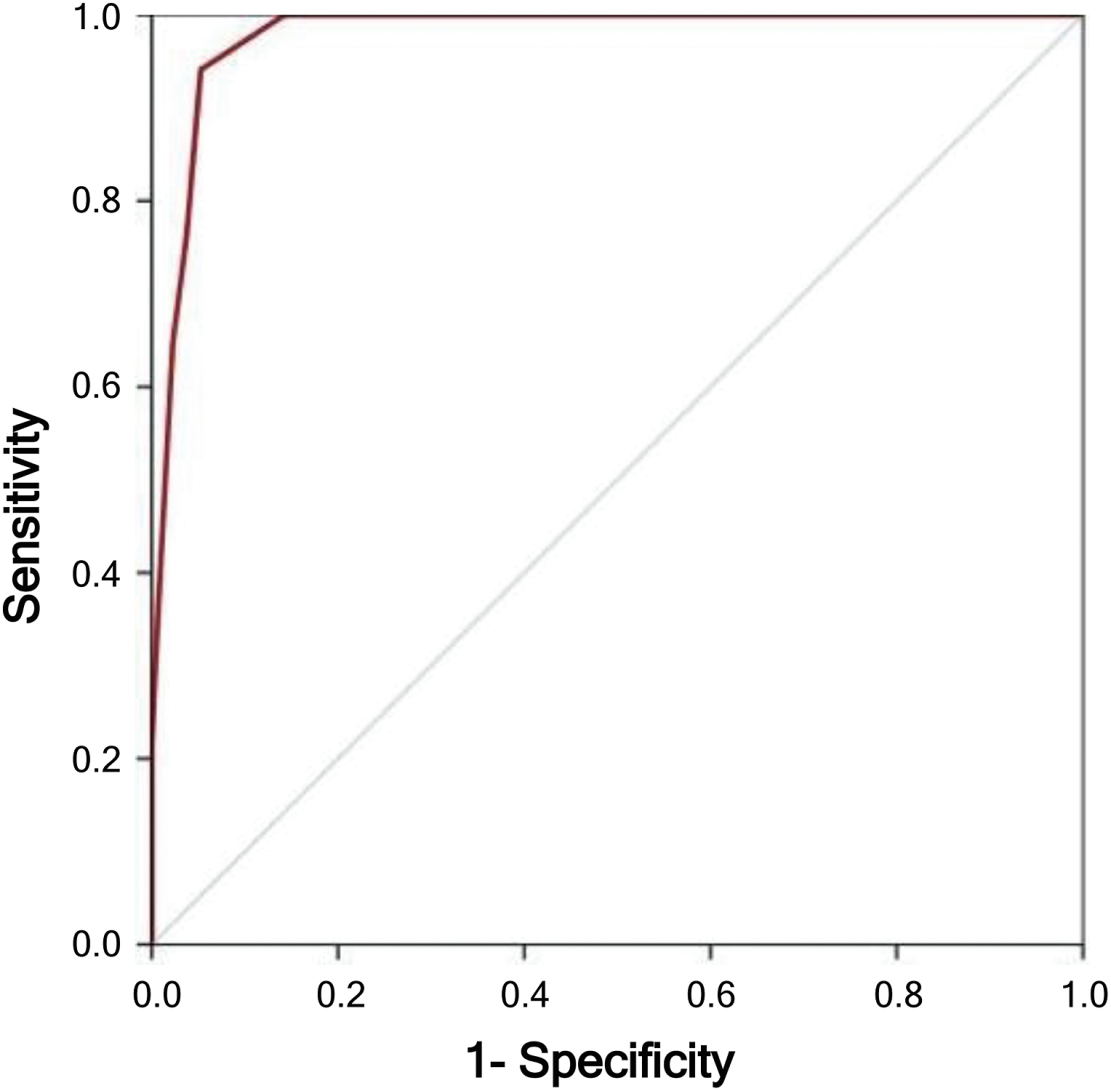
Receiver operating characteristic (ROC) curve for the Turkish version of rapid eye movement sleep behavior disorder (RBD) symptom score. Area under the curve (AUC) = 0.978 (95% confidence interval [95%CI]: 0.960–0.996; *p* < 0.001).

The Cronbach's α coefficient for the symptom section of the IRBD-9-TR was of 0.857. The agreement between the IRBD-9-TR and the RBDSQ-T was demonstrated through the Kappa coefficient of 0.885 ( *p* < 0.001). The RBDSQ-T score and the the symptom score on the IRBD-9-TR were highly correlated (r = 0.893; *p* < 0.001) ( Figure 1 ).

### Criterion validity

**Figure 2:**
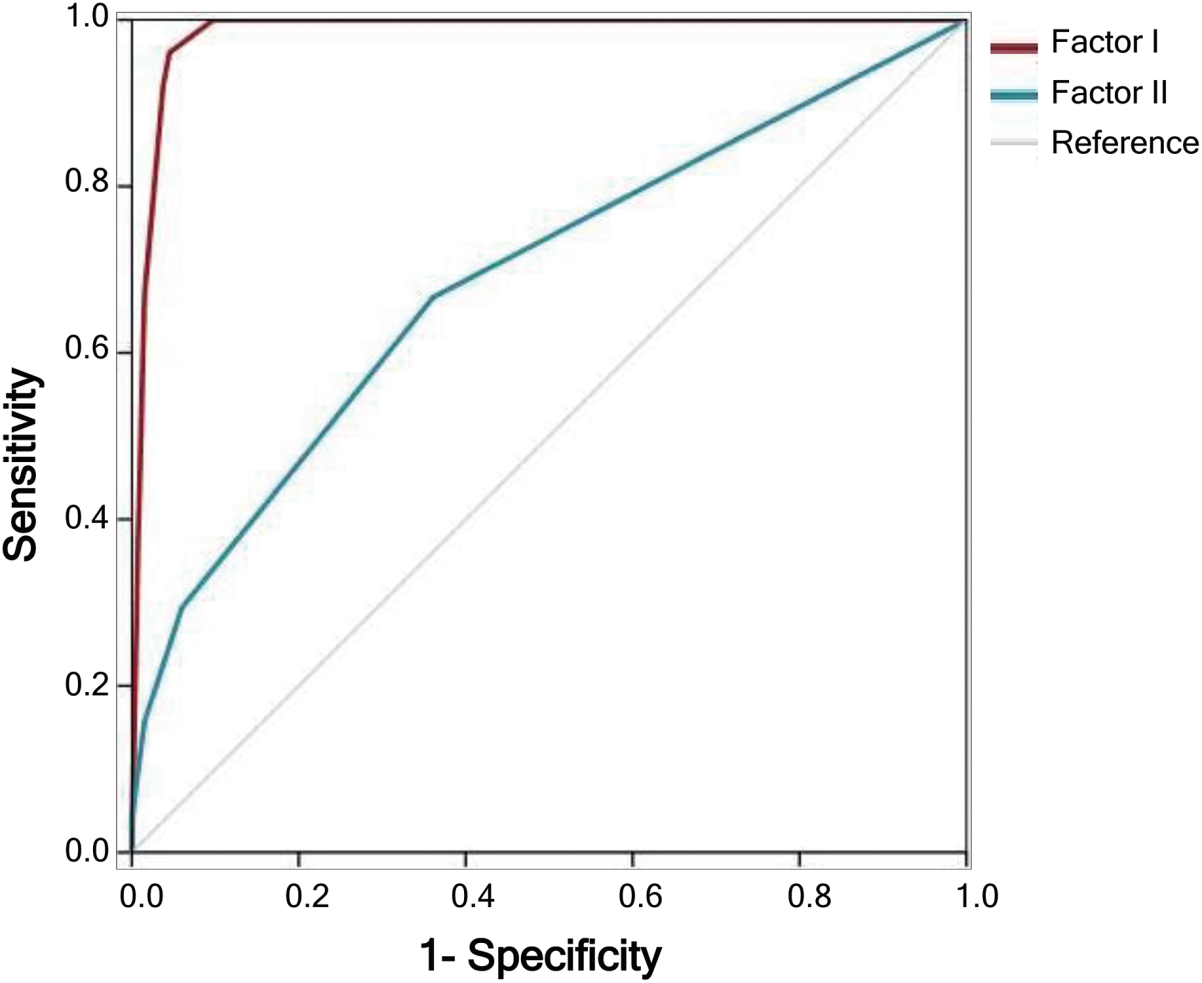
Graph illustrating the ROC curve and separating the group of RBD subjects from the group of healthy subjects using the symptom score from factors I and II.

The optimal cut-off value for the IRBD-9-TR symptom score was of 0.28, with a sensitivity of 0.941 and a specificity of 0.947. Accordingly, 94.4% of the patients were correctly diagnosed ( Table 5 ). For Factor I, the AUC was of 0.984 (95% confidence interval [95%CI]: 0.968–1.000; *p* < 0.001), and, for Factor II, it was of 0.689 (95%CI: 0.598–0.779; *p* < 0.001) ( Figure 2 and Table 6 ).

**Table 5:** Diagnostic performance of the IRBD-9-TR regarding RBD patients and healthy subjects

In comparison to the RBD	Cut-off	Sensitivity (%)	Specificity (%)	PPV (%)	NPV (%)	LR (+)	LR (-)	AUC	*p*
Healthy subjects	0.278	94.1	94.7	87.3	97.7	17.9	0.06	0.98	< 0.001

Abbreviations: RBD, rapid eye movement sleep behavior disorder; IRBD-9-TR, Turkish version of the Innsbruck Rapid Eye Movement Sleep Behavior Disorder Diagnostic Inventory; PPV, positive predictive value; NPV, negative predictive value; LR(+), positive likelihood ratio; LR(-), negative likelihood ratio; AUC, area under the curve.

**Table 6:** Diagnostic performance of the symptom section of the IRBD-9-TR for factors I and II

RBD patients and healthy subjects	Cut-off	Sensitivity (%)	Specificity (%)	PPV (%)	NPV (%)	LR (+)	LR (-)	AUC	*p*
**Factor I**	0.300	96.1	95.5	89.1	98.5	21.3	0.04	0.98	<0.001
**Factor II**	0.125	66.7	63.9	41.5	83.3	1.85	0.52	0.69	<0.001

Abbreviations: AUC, area under the curve; IRBD-9-TR, Turkish version of the Innsbruck Rapid Eye Movement Sleep Behavior Disorder Diagnostic Inventory; LR(-), negative likelihood ratio; LR(+), positive likelihood ratio; NPV, negative predictive value; PPV, positive predictive value; RBD, rapid eye movement sleep behavior disorder.

## DISCUSSION

The present study demonstrated that the IRBD-9-TR presents strong validity and reliability. The optimal cut-off value for the symptom score was of 0.28. The diagnostic accuracy of each individual question showed that questions 1, 2, 3, 6, and 8 (factor I) presented higher specificity than questions 4, 5, 7, and 9 (factor II). On the other hand, the factor-I questions presented excellent discrimination. We also observed that the short version of the IRBD-9-TR presented strong validity and reliability, which is in line with the study by Frauscher et al. 13 In the short form, the cutoff value (0.28) was very close to the value (0.25) of the original form, and the sensitivity (0.941) and specificity (0.947) were a little high than original form. With the application of the short form of the IRBD-9-TR, 94.4% of the patients included in the current study were correctly diagnosed. The original form of the IRBD-9-TR was thought to be prioritized in terms of diagnosing and monitoring progression in iRBD patients, because it was easy to use in clinical settings, it contained fewer questions, and it aimed to determine the frequency of symptoms.

Frauscher et al. 13 also reported that the IRBD-9 scores were not different among patients with iRBD and those with symptomatic RBD. 13 In the ICSD-3, the diagnostic criteria for RBD do not differentiate it as symptomatic or idiopathic. 4 As stated by the authors of the original article (Frauscher et al), both in iRBD and symptomatic RBD groups, the clinical signs and symptoms of the patients are very similar or the same at the beginning. However, it has been suggested that the duration of RSWA in iRBD patients is an electrophysiologic marker of phenoconversion. 20 In its original form, the IRBD-9 was found to be highly specific and sensitive in determining the diagnosis of RBD in both idiopathic and symptomatic RBD patients, although it did not differentiate the group, but the RBDSQ has been shown 21 to be effective in diagnosing RBD in the general population, but not sensitive enough to identify RBD in neurological diseases such as PD. The most important difference between the present study and the one by Frauscher et al. 13 and perhaps other studies 9 13 22 is that we did not have a group of symptomatic RBD patients, the subjects were not informed about the diagnosis of RBD, and the tests were conducted before the vPSG was reported and the patient was informed.

The limitation of the current study is that we did not include symptomatic RBD patients and did not compare the results of IRBD-9-TR scores between iRBD and symptomatic RBD patients. On the other hand, we could not evaluate the ability of the short form of the IRBD-9-TR to distinguish between symptomatic RBD and iRBD patients.

The strengths of ther present study are that the patients were not informed about their diagnosis before the vPSG and the screening test. The fact that the study was conducted at multicentric accredited sleep centers showed that this screening test can be easily used in sleep centers.

In conclusion, we demonstrated that the IRBD-9-TR and its short form present high specificity and sensitivity to be used in the clinical practice. In addition, it can be used in further epidemiologic studies in the general population, and in other studies analyzing iRBD patients in Turkey, such as studies investigating phenoconversion after the diagnosis of iRBD. Lastly, the use of the IRBD-9-TR should be further investigated in other populations, such as in patients with neurodegenerative disorders, or in patients with symptomatic RBD.
